# Epidemiology of Concomitant Infection Due to *Loa loa* and *Mansonella perstans* in Gabon

**DOI:** 10.1371/journal.pntd.0001329

**Published:** 2011-10-11

**Authors:** Jean Paul Akue, Dieudonné Nkoghe, Cindy Padilla, Ghislain Moussavou, Hubert Moukana, Roger Antoine Mbou, Benjamin Ollomo, Eric Maurice Leroy

**Affiliations:** 1 Department of Medical Parasitology, Centre International de Recherches Médicales de Franceville (CIRMF), Franceville, Gabon; 2 Unité des Maladies Virales Emergentes, Centre International de Recherches Médicales de Franceville, Franceville, Gabon; 3 Ministry of Health, Libreville, Gabon; 4 Department of Immunodeficiency and Infectious Diseases, University of Liege, Liege, Belgium; 5 MIVEGEC (IRD 224/CNRS 5290/UM1/UM2), Montpellier, France; London School of Hygiene and Tropical Medicine, United Kingdom

## Abstract

**Background:**

The filarial parasites *Loa loa* and *Mansonnella perstans* are endemic in the central and western African forest block. *Loa loa* is pathogenic and represents a major obstacle to the control of co-endemic filariae because its treatment can cause fatal complications such as encephalitis.

**Methodology/Principal Findings:**

4392 individuals aged over 15 years were studied both by direct examination and a concentration technique. The overall prevalence rates were 22.4% for *Loa loa* microfilaremia, 10.2% for *M. perstans* microfilaremia, and 3.2% for mixed infection. The prevalence of both filariae was higher in the forest ecosystem than in savannah and lakeland (p<0.0001). The intensity of microfilariae (mf) was also higher in the forest ecosystem for both parasites. The prevalence and intensity of microfilaria were both influenced by age and gender. Correlations were found between the prevalence and intensity of *Loa loa* microfilariae (r = 0.215 p = 0.036), and between the prevalence of *Loa loa* and the prevalence of individuals with microfilaria >8000 mf/ml (r = 0.624; p<0.0001) and microfilariae >30 000 mf/ml (r = 0.319, p = 0.002). In contrast, the prevalence of pruritis and Calabar swellings correlated negatively with the prevalence of *Loa loa* microfilaria (r = −0.219, p = 0.032; r = −0.220; p = 0.031, respectively). Pruritis, Calabar swellings and eye worm were not associated with *L. loa* mf intensity (r = −0.144, p = 0.162; r–0.061, p = 0.558; and r = 0.051, p = 0.624, respectively), or with the prevalence or intensity of *M. perstans* microfilariae.

**Conclusions/Significance:**

This map of the distribution of filariae in Gabon should prove helpful for control programs. Our findings confirm the spatial uniformity of the relationship between parasitological indices. Clinical manifestations point to a relationship between filariae and allergy.

## Author Summary

*Loa loa* and *Mansonella perstans* are blood filarial parasites, endemic in the central and western African forest block, and transmitted by chrysops and culicoides flies, respectively. *Loa loa* is pathogenic and represents a major obstacle to the control of co-endemic filariae. Treatment of individuals with >8000 *Loa loa* microfilariae/ml can result in severe adverse reactions. *M. perstans* is prevalent in the tropics, with undefined clinical symptoms. We screened 4392 individuals for these infections in 212 Gabonese villages. The overall prevalence rates were 22.4% for *Loa loa* microfilariae, 10.2% for *M. perstans*, and 3.2% for mixed infection. These rates varied across the different ecosystems: forest, savannah, Lakeland, river (Ogouée), and equator. A correlation was found between the prevalence and intensity of microfilariae, while a negative relationship was found between clinical symptoms (pruritis, Calabar swelling) and the prevalence of *Loa loa* microfilaremia. This study confirms the spatial uniformity of the relationship between parasitological indices, and provides a map and baseline data for implementation of mass chemotherapy for these infections.

## Introduction

*Loa loa* and *Mansonella perstans* are endemic filarial parasites in the central and western African rainforest. *Loa loa* infects 2 to 3 million people [1]. *M. perstans* is considered non pathogenic [2]–[3], although some clinical manifestations have been associated with *M. perstans* microfilaria [4], [5], [6] including ocular disorders [7], [8]. Interest in loiasis has grown during the last 30 years, for several reasons. First, in endemic areas loiasis is the second reason for medical visits, after malaria [1], [9]. Second, this infection mainly affects active young individuals, who contribute to agricultural productivity [10], and their health is often aggravated by co-infection by other parasites. Two-thirds of infected individuals are amicrofilaremic, despite subconjunctival migration of adults worms, suggesting immunological elimination of microfilariae [1], [11]. Severe adverse events can occur during treatment with diethylcarbamazine (DEC) and ivermectin in individuals with high-level microfilaremia, requiring close treatment monitoring and hindering mass administration of antifilarial drugs aimed at controlling other filariae in areas where *Loa loa* is co-endemic. This is not the case with *M. perstans* [12].

Many epidemiological studies of loiasis and Mansonellosis have been carried out throughout the western and central African forest block. These studies mainly focused on the distribution of loiasis and on the possible relationship between the prevalence and intensity of microfilaremia, in order to estimate the risk of adverse events during mass chemotherapy.

The prevalence of *L. loa* microfilaremia varies from country to country [13], as well as within a given country and even a given geographic area [14]. The highest prevalence is observed in forest areas and the lowest in savannah areas of both Gabon [15], [16] and Cameroon [17], [18], for example. Differences within a given geographic zone are directly linked to the bioecological specificity of a microzone [19]. These observations were recently used to create a predictive geographical model of loiasis endemicity based on satellite, vector habitat, prevalence, vegetation, temperature, relief, pluviometry and topography data [20]. However, when compared to field data, this model showed certain limitations [21].

A linear relationship between the prevalence and intensity of loiasis has been established. A high prevalence is indicative of intense *L. loa* infection and therefore a high risk of adverse events [22], [23]. The 20% threshold prevalence of microfilaremia at the community level corresponds to about 5% of high microfilaremia loads (>8000 mf/ml) and 2% of very high microfilaremia loads (>30000 mf/ml), the latter being the cut-off point above which there is a risk of severe adverse events during ivermectin treatment [24]. Owing to the difficulties of drawing regional maps based on microscopic analysis, a rapid method for evaluating the prevalence and intensity of *Loa loa* infection at the community level has been developed (RAPLOA: Rapid Assessment of Prevalence of *Loa loa*) [25]. RAPLOA is based on interviews assisted by photographs of adult worms in the eye, to detect subconjunctival migration of adult worms (which lasts 1 to 7 days), as reported by interviewees. A 40% prevalence of a history of eye worm corresponds to a 20% threshold prevalence of microfilaremia at the community level, 5% of high microfilaremia loads (>8000 mf/ml) and 2% of very high microfilaremia loads (>30000 mf/ml) [25]. Another clinical manifestation, Calabar swellings, was used to evaluate the risk of adverse events. This sign has shown to correlate with the prevalence of highly microfilaremic individuals [25].

The use of eye worm and Calabar edema to assess the risk of fatal side effects in patients with loiasis suggests a relationship between clinical symptoms and parasitological indices.

Most of these latter studies were performed in Cameroon, Nigeria, Republic of Congo and Democratic Republic of Congo [17]–[25], only a few concerning Gabon.

In Gabon, epidemiological surveys have identified five filarial species (*L. loa*, *M. perstans*, *O. volvulus*, *M. streptocerca*, and *M. rodhaini*), and yielded a preliminary map [12]–[16], [26]–[28]. *L. loa* is the predominant species and co-exists with *M. perstans*. The prevalence of microfilaremia varies across provinces and even within a given province, being higher in mountain forest than in savannah.

The aim of the present study was to obtain a fuller picture of the distribution of blood-borne filariae in Gabon, using both the wet blood film and concentration techniques, and to detect a linear relationship between the prevalence and intensity of loiasis and between clinical symptoms and parasitological indices. We therefore conducted a large survey, including all the country's ecological niches and recording the main clinical manifestations of *Loa loa* infection.

## Materials and Methods

### Area of study

**Figure 1:**
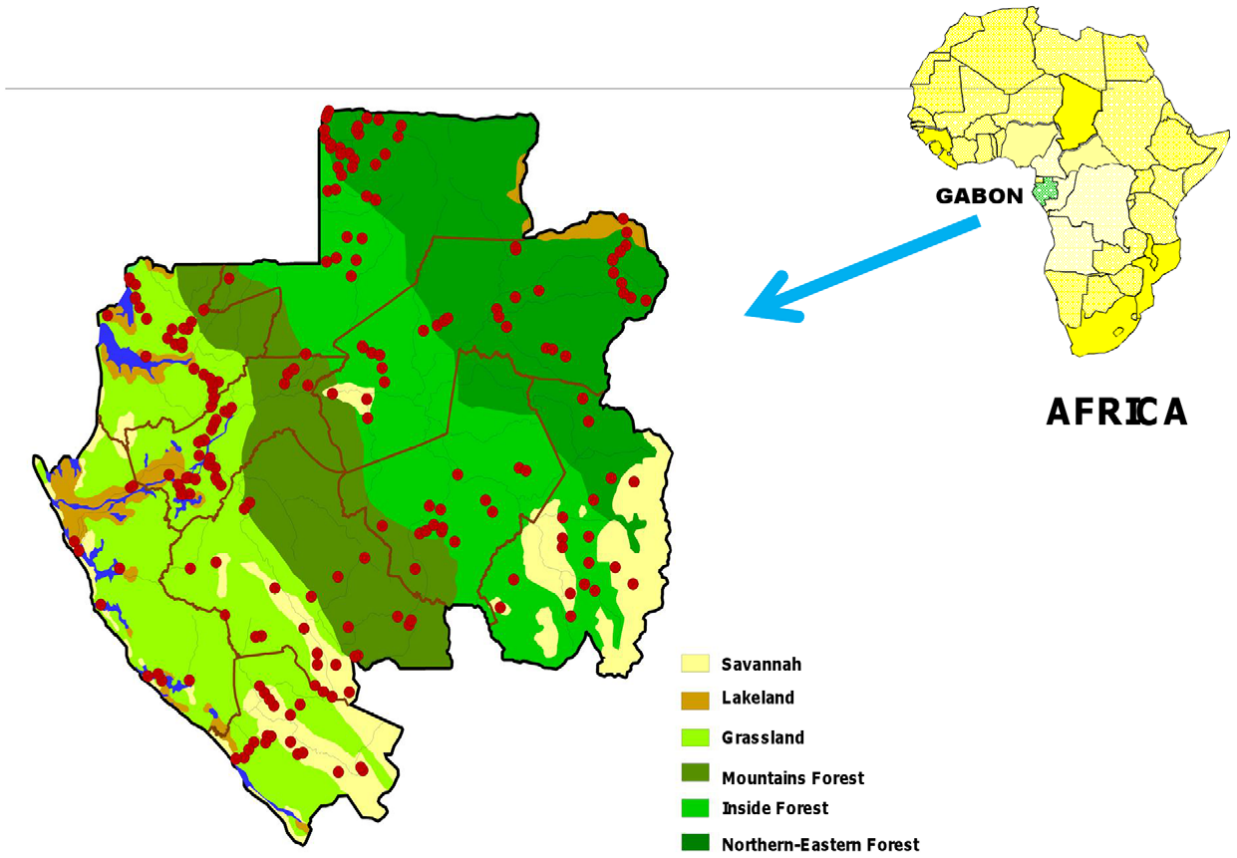
Map of Gabon with administrative regions and the locations of surveyed villages (red circles). Strictly georeferenced and generated by MAPINFO. The ecosystems are represented in different colours.

We surveyed rural Gabonese populations. The country is 800 km long and 20 to 300 km wide, consists of 80% rain forest, and is bordered to the west by the Atlantic Ocean. The forest zone extends from west to east, from the coastal basin with the grassland forest to the interior and north-eastern forest plates band, through a wide mountainous forest band from 60 to 100 km parallel to the coast. The south and southeast contain isolated areas of *savannah* and *steppe*. A coastal and continental marine ecosystem named *lakeland* is located around the mouth of River Ogooué (Figure 1) [29]. The population is about 1.5 million and there are 2048 villages located in 9 provinces. Rural populations are located along roads and rivers, and few villages have more than 300 inhabitants.

### Study population

This survey was conducted during nine-month field missions between June 2005 and September 2008. For this survey, a stratified random sampling method was used, based on the 9 provinces. Twenty to 30 villages per province were randomly selected. The required sample size was calculated on the basis of an estimated prevalence of 5 to 10% (using n = ε^2^ [p (1−p)]/e^2^; with ε = 1.96 (alpha risk = 5%), e (precision) = 2% and p = expected prevalence; with n varying from 188 to 864). Within each village, individuals over 16 years of age having lived for at least one year in their village and who accepted blood sampling where included in the study. A free medical examination was offered and basic medicines were provided to all participants and non participants, if appropriate. All the villages were georeferenced (Figure 1).

### Questionnaire

The rationale of the study was explained and a one-page questionnaire was administered to all participants. We collected demographic data (age, sex and occupation), geographic data (name of the village, length of residence, department and province) and the medical history (eye worm, Calabar swellings, chronic arthralgia, pruritus, etc.) (Figure S1).

### Ethical considerations

The study protocol was approved by the Ministry of Health. The Health Director, the governor of each province and the chiefs of each village received written information. Individual written consent was required before blood sampling. The results of the study were transmitted to the Ministry of Health.

### Blood collection

Field laboratory facilities were set up in regional hospitals. Blood samples were collected, usually in the villages' healthcare centers, on a daily basis, into two 7-ml Vacutainer tubes containing EDTA (VWR International, France). The tubes were stored in the dark at +4°C before transportation to the field laboratory.

### Parasitological analyses

Due to the variability of microfilarial load, the analysis started systematically by direct examination of a wet blood film, followed by a concentration technique. Two experienced technicians read the slides separately, and the results were controlled by a parasitologist. Briefly, microfilariae were counted directly in a 10-µl wet blood film between microscope slide and coverslip, using an optical microscope equipped with a 10× objective. Parasitemia was expressed in microfilariae per milliliter (mf/ml) of blood. A modified Knott's concentration technique [30] was applied routinely to each sample, as follows: 1 ml blood was diluted with 9 ml PBS in a conical tube and 200 µl of saponin (2% w/v) was added to lyze red cells. The tubes were centrifuged (10 min, 500 *g*) and the supernatants discarded. The entire pellet was then examined under the microscope (10× objective) and microfilariae were counted. Parasite species were identified by their size and motility, and by the absence or presence of a sheath.

### Data analysis

*Loa loa* prevalence rates were estimated nationwide. As mentioned above, the 20% threshold prevalence of microfilaremia is the cut-off above which serious adverse events are likely to occur, and corresponds to 5% of high microfilaremia loads (>8000 mf/ml) and 2% of very high microfilaremia loads (>30000 mf/ml). Thus, prevalence rates were calculated in each province, village and ecosystem as prevalence rates for microfilaremia loads >8000/ml and >30000/ml. The intensity of infection was estimated as described elsewhere [25]. The difference between the results of the two laboratory tests was calculated. The Chi2 test and Fisher's exact test were used as appropriate. Minitab 16 software was used to calculate Spearman's correlation coefficient for the association between parasitological and clinical parameters, and the Mann Whitney U test was used to compare mf intensity among groups. Univariate crude conditional maximum likelihood estimates of odds ratios (OR) and exact 95% confidence intervals (CI) were determined for each potential risk factor, using STATA software version 9.0 (Stata Corporation, College Station, USA). Multivariate logistic regression models stratified by the ecosystem were constructed from risk factors with a significance of ≤0.10 in univariate analysis, using a backwards stepwise elimination procedure. P values below 0.05 were considered statistically significant.

## Results

### Characteristics of the study population

In total, 4392 individuals from 15 to 85 years old were enrolled in 212 villages, representing 10.7% of all villages in the country. The distance between villages ranged from 5 to 30 km. The sex ratio (M/F) was 0.88 (47.4% men and 52.6% women). About 58% of individuals were more than 45 years old and 63.9% had spent more than 10 years in their village. Farmers represented 69.8% of the population and hunters 10.2%. Around 80% of individuals were surveyed in the forest area, 10% in the savannah and the lakeland. The reported proportions of eye worm, Calabar swellings and pruritis were 29.3%, 11.2% and 22.4% (Table 1).

**Table 1:** Sociodemographic and clinical characteristics of the study population.

Characteristics		Total number	Percentage %
Sex	Men	2084	47.4
	Women	2308	52.6
Age	[15–30[	645	14.7
	[30–45[	1146	26.1
	[45–60[	1421	32.4
	≥60	1180	26.9
Length of residence	<10 years	1514	36.1
	>10 years	2682	63.9
Occupation	Farming	3067	69.8
	Hunting	448	10.2
	Others	680	15.5
	Unknown	197	4.5
Location	Forest	3478	79.2
	Savannah	460	10.5
	Lakeland	454	10.3
Clinical examination	Eye worm	941	29.3
	Calabar swellings	353	11
	Pruritus	1229	29.4

### Comparison of microfilariae counts with and without concentration

The wet blood smear identified 790 *Loa loa* and 116 *M. Perstans* microfilaremic subjects while the concentration technique detected 984 *L. loa* and 447 *M. perstans* microfilaremic subjects (difference of 19.7% for *L. loa* and 74% for *M. perstans*) (Table 2).

**Table 2:** Comparison of wet blood smear and the concentration technique for the detection of the filariae.

Filarial species	Direct examination	Leukoconcentration	Difference n (%)
***Loa loa***	790	984	194 (19.7)
***Mansonella perstans***	116	447	331(74)
**TOTAL**	**906**	**1431**	**525 (36.6)**

Most of these individuals who were positive only after concentration had microfilaremia below 100/ml, for both species (Table 3).

**Table 3:** Evaluation of the concentration technique in individuals with different levels of microfilaremia.

Filarial species	Microfilaremia <100/ml	Microfilaremia <200/ml	Difference n (%)
***Loa loa*** ** n = 194**	179	192	13 (6.7)
***Mansonella perstans*** ** n = 447**	435	444	9 (2)
**TOTAL n = 641**	**614**	**636**	**22 (3.4)**

### Geographic distribution of *L. loa* and *M. perstans* microfilaremia

**Figure 2:**
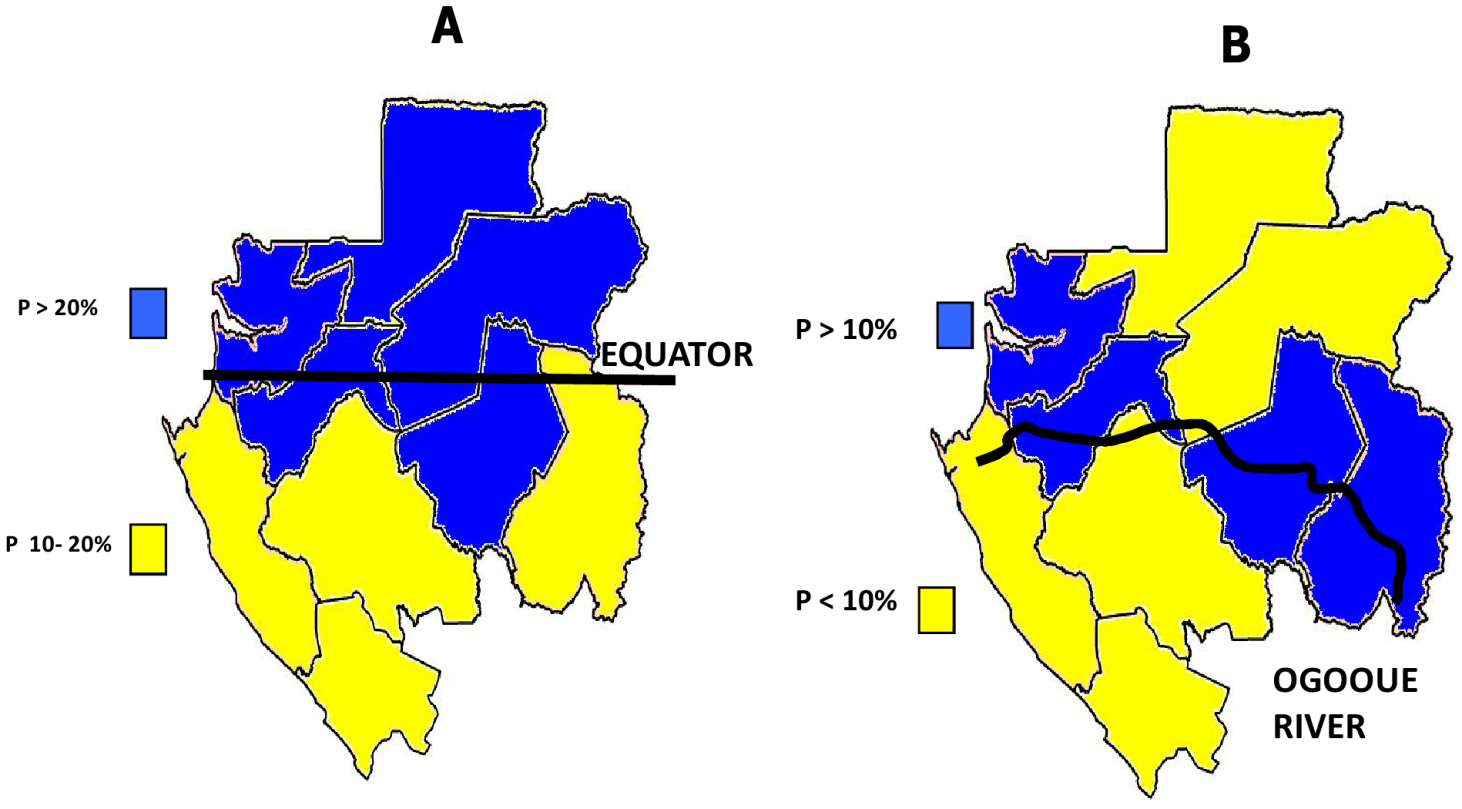
Distribution of *Loa loa* (A) and *Mansonella perstans* (B) in Gabon according to the geographic region.

The overall prevalence rates of *L. loa* and *M. perstans* microfilaria were respectively 22.4% (95%CI: 21.2–23.7) (up to 57% in some villages), and 10.2% (95%CI: 9.3–11.1) (up to 67% in some villages), while 3.2% of subjects were coinfected (95%CI: 2.7–3.8) (Table 4, Table S1). The highest prevalence was found in the North Equator region (Figure 2A) for *Loa loa* (>10–20%) and along the Ogooué river for *M. perstans* (Figure 2B).

**Table 4:** Prevalence of *Loa loa* and *Mansonella perstans* microfilaremia in the nine administrative regions of Gabon.

Provinces	Sampling period	Number of villages surveyed	*Loa loa*			*Mansonella perstans*			Co- infection		
			+/Total	%	CI 95%	+/Total	%	CI 95%	+/Total	%	CI 95%
ESTUAIRE	July 2005	30	105/314	33.4	28.3–39	72/314	22.9	18.5–28.1	29/314	9.2	6.4–13.1
HAUT OGOOUE	April 2007	18	66/364	18.1	14.4–22.6	48/364	13.2	10–17.2	9/364	2.5	1.2–4.8
MOYEN OGOOUE	January 2006	31	159/603	26.4	22.9–30.1	88/602	14.6	11.9–17.8	32/602	5.3	3.7–7.5
NGOUNIE	June 2006	22	86/463	18.6	15.2–22.5	43/461	9.3	6.9–12.4	14/461	3	1.7–5.2
NYANGA	January 2007	16	76/426	17.8	14.4–21.9	12/425	2.8	1.5–5	6/425	1.4	0.6–3.2
OGOOUE IVINDO	June 2007	41	153/624	24.5	21.2–28.1	35/624	5.6	4–7.8	14/624	2.2	1.3–3.8
OGOOUE LOLO	September 2007	18	118/423	27.9	23.7–32.5	81/423	19.1	15.6–23.3	23/423	5.4	3.6–8.2
OGOOUE MARITIME	May 2008	10	25/206	12.1	8–17.4	3/206	1.4	0.3–4.2	1/206	0.5	0–2.7
WOLEU NTEM	April 2006	34	196/969	20.2	17.8–22.9	65/969	6.7	5.3–8.5	13/969	1.3	0.7–2.3
**TOTAL**		220	**984/4392**	**22.4**	**21.2–23.7**	**447/4388**	**10.2**	**9.3–11.1**	**141/4388**	**3.2**	**2.7–3.8**

In the administrative regions, Estuaire province had the highest prevalence of *L. loa* (33.4%), *M. perstans* (22.9%) and co-infection (9.5%), while Ogooue maritime province had the lowest prevalence rates (respectively 12.1%, 1.4% and 0.5%) (Table 4).

**Figure 3:**
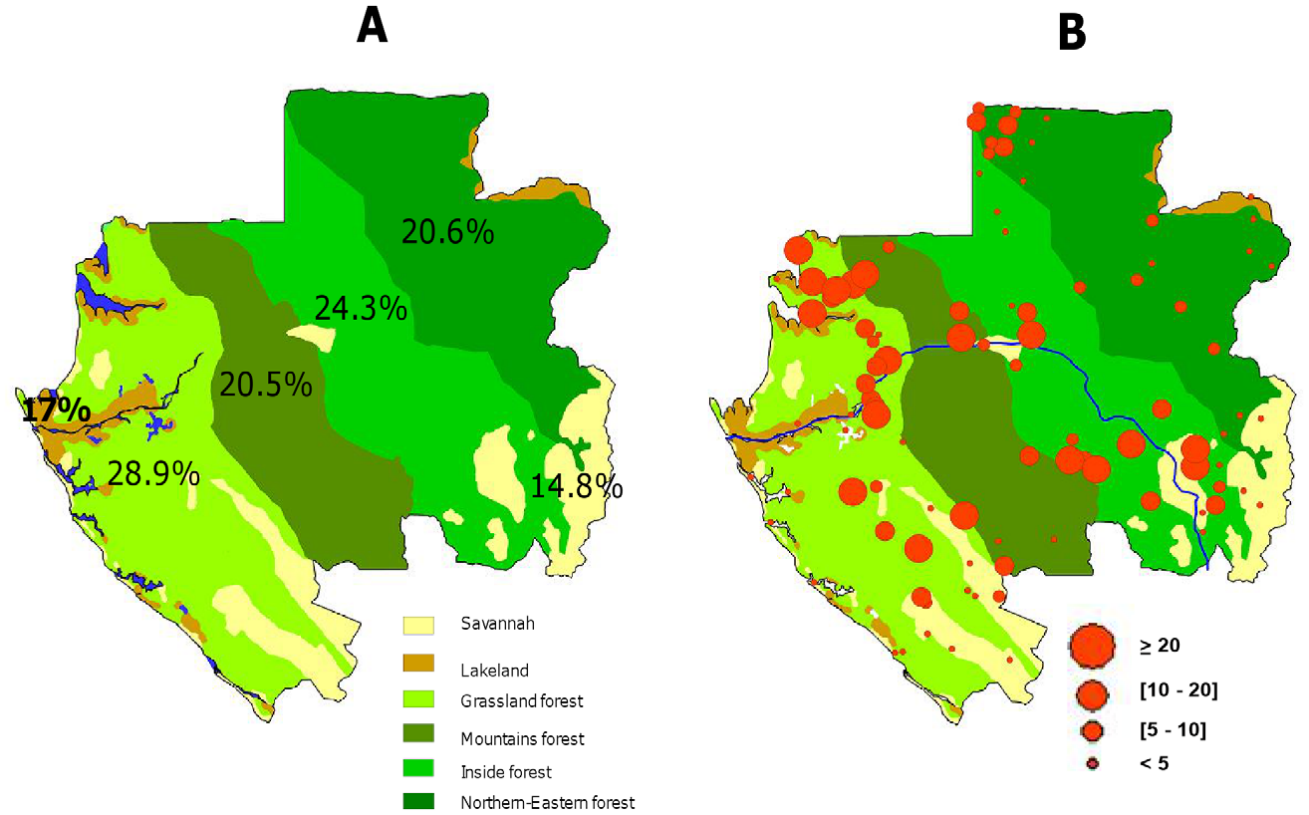
Distribution of *L. loa* in Gabon in the different ecosystems (A) (Prevalence rates of *Loa loa* are shown within the corresponding ecosystem), and villages (B).

In the ecological regions, the *L. loa* prevalence rate (Table 5, Figure 3A) was significantly higher (p<0.0001) in the forest (24.1%) than in the lakeland (17%) and savannah (14.8%). No difference (p = 0.4) was observed between lakeland and savannah. Moreover, within the forest ecosystem, the prevalence was significantly higher in grassland (28.9%) than in the mountain (20.5%), interior (24.3%) and north eastern (20.6%) forest regions (p<0.0002). In the same way, the *M. perstans* prevalence rate (Table 5) was significantly higher (p<0.0001) in the forest region (11.3%) than in lakeland (4.2%) and savannah (7.4%), and no difference (p = 0.053) was observed between lakeland and savannah. Within the forest ecosystem, the prevalence in the north-eastern forest (5.2%) was significantly lower (p<0.0001) than in the grassland (14.6%), mountain (14.9%) and interior forest (11.9%) (Table 5). Finally, most villages with high *L. loa* prevalence rates were located in the forest area (Table S1, Figure 3B).

**Table 5:** Prevalence of *Loa loa* and *Mansonella perstans* microfilaremia in the main ecosystems of Gabon.

	Number of villages surveyed	*Loa loa*			*Mansonella perstans*		
Ecosystems		Positive/tested	% (95% CI)	p value	Positive/tested	% (95% CI)	p value
**Lakeland**	24	77/454	17 (13.7–20.8)	<0.0001	19/454	4.2 (2.6–6.6)	<0.0001
**Savannah**	22	68/460	14.8 (11.7–18.4)		34/460	7.4 (5.2–10.3)	
**Forest**	174	839/3478	24.1 (22.7–25.6)		394/3474	11.3 (10.3–12.5)	
*Grassland forest*	62	258/894	28.9 (25.9–32)	<0.0002	130/892	14.6 (12.4–17.1)	<0.0001
*Mountains forest*	22	87/425	20.5 (16.8–24.7)		63/423	14.9 (11.7–18.7)	
*Inside forest*	50	322/1326	24.3 (22–26.7)		158/1326	11.9 (10.2–13.8)	
*North eastern forest*	40	172/833	20.6 (18–23.6)		43/833	5.2 (3.8–6.9)	
***All population***	***220***	***984/4392***	***22.4 (21.2–23.7)***		***447/4392***	***10.2 (9.3–11.1)***	

### Analysis of risk factors

In univariate analysis, males had a significantly higher risk of *Loa loa* infection than females (OR: 2.38, 95%CI: 2.05–2.75, p<00001), and the prevalence of *Loa loa* parasitemia increased linearly with age (p<0.00001) (Table 6). The prevalence of *Loa loa* microfilaremia was higher in hunters than in farmers and other occupational groups (p<0.04), and higher in individuals with eye worm (p<0.001) and those without Calabar swellings (p<0.014) (Table 6). Only gender was a risk factor for *M. perstans* microfilaremia, males having a significantly higher prevalence than females (OR: 1.89, 95%CI: 1.54–2.31, p<0.0001) (Table 7). In multivariate analysis, only age and sex remained significantly associated with *Loa loa* parasitemia, throughout the country and within the forest ecosystem (Table 8 and 9).

**Table 6:** Univariate analysis of sociodemographic and clinical risk factors for *Loa loa* microfilaremia in Gabon.

Variable			Number (%)	95% CI	OR[95%CI]	p-value
**Sex**	Men		629 (30.2)	28.2–32.2	2.38 [2.05; 2.75]	<0.0001
	Women		355 (15.4)	13.9–16.9	1	
**Age**	[15–30[		94 (14.6)	11.8–17.3	1	<0.0001
	[30–45[		234 (20.4)	18.1–22.7	1.50 [1.16; 1.95]	
	[45–60[		348 (24.5)	22.2–26.7	1.90 [1.48; 2.44]	
	≥60		308 (26.1)	23.6–28.6	2.07 [1.60 ; 2.67]	
**Occupation**	Farming		657 (21.4)	20–22.9	0.77 [0.61 ; 0.97]	0.04
	Hunting		117 (26.1)	22.2–30.5	1	
	Others		165 (24.3)	21.1–27.7	0.91 [0.69 ; 1.19]	
**Clinical examination**	Eye worm	Yes	232 (24.7)	22–27.6	1.34 [1.12 ; 1.61]	0.001
		No	445 (19.6)	18–21.3	1	
	Calabarswellings	Yes	56 (15.9)	12–19.7	0.69 [0.51 ; 0.93]	0.014
		No	613 (21.5)	20–23	1	
	Pruritus	Yes	259 (21.1)	18.8–23.4	0.89 [0.76; 1.05]	0.155
		No	682 (23.1)	21.6–24.6	1	

**Table 7:** Univariate analysis of sociodemographic and clinical risk factors for *Mansonella perstans* microfilaremia in Gabon.

Variable			Number (%)	95% CI	OR[95%CI]	p-value
Sex	Men		275 (13.2)	11.7–14.7	1.89 [1.54; 2.31]	<0.0001
	Women		172 (7.5)	6.4–8.5	1	
Age	[15–30]		55 (8.5)	6.4–10.7	1	0.197
	[30–45]		118 (10.3)	8.5–12.1	1.23 [0.88; 1.72]	
	[45–60]		138 (9.7)	8.2–11.3	1.15 [0.83; 1.60]	
	≥60		136 (11.5)	9.7–13.4	1.40 [1.01 ; 1.95]	
Occupation	Farming		296 (9.7)	8.6–10.7	0.75 [0.55 ; 1.01]	0.168
	Hunting		56 (12.5)	9.4–15.6	1	
	Others		71 (10.4)	8.1–12.7	0.82 [0.56 ; 1.18]	
Clinical examination	Eye worm	Yes	86 (9.15)	7.3–11	1.23 [0.94 ; 1.61]	0.138
		No	172 (7.6)	6.5–8.7	1	
	Calabarswellings	Yes	28 (7.93)	5.1–10.8	0.99 [0.66 ; 1.5]	0.998
		No	226 (7.94)	6.9–8.9	1	
	Pruritus	Yes	110 (8.95)	7.4–10.5	0.82 [0.65; 1.03]	0.092
		No	315 (10.7)	9.6–11.8	1	

**Table 8:** Multivariate analysis of sociodemographic and clinical risk factors for *Loa loa* microfilaremia in Gabon.

Variable		OR	[95%CI]	p-value
**Sex**	Men/Women	2.07	1.70–2.52	<0.0001
**Age**	[15–30[	1		
	[30–45[	1.19	0.86–1.65	0.287
	[45–60[	1.75	1.28–2.38	<0.0001
	≥60	1.76	1.28–2.41	<0.0001
**Occupation**	Farming	0.94	0.73–1.21	0.614
	Hunting	1		
	Others	0.83	0.60–1.14	0.244
**Clinical examination**	Eye worm	1.42	1.17–1.73	<0.0001
	Calabar swellings	0.68	0.49–0.95	0.022
	Pruritus	0.98	0.81–1.20	0.876

**Table 9:** Multivariate analysis of sociodemographic and clinical risk factors for *Loa loa* microfilaremia in forest ecosystem.

Variable		OR	[95%CI]	p-value
**Sex**	Men/Women	2.07	1.68–2.55	<0.0001
**Age**	[15–30[	1		
	[30–45[	1.21	0.85 –1.72	0.282
	[45–60[	1.79	1.29–2.50	<0.0001
	≥60	1.80	1.28–2.51	<0.0001
**Occupation**	Farming	0.92	0.70–1.21	0.564
	Hunting	1		
	Others	0.82	0.57–1.15	0.250
**Clinical examination**	Eye worm	1.42	1.16–1.74	0.001
	Calabar swellings	0.61	0.43–0.87	0.006
	Pruritus	1.00	0.81–1.24	0.996

For clinical symptoms, only eye worm and Calabar swellings remained significantly associated with *Loa loa* parasitemia, both throughout the country and within the forest ecosystem (Table 8 and 9).

### Intensity of microfilaremia

Microfilaremia in *Loa loa*-positive individuals ranged from 1 to 500 000 mf/ml (arithmetic mean: 5441 mf/ml; median: 900 mf/ml), while *M. perstans* microfilaremia ranged from 1 to 12 000 mf/ml (mean: 189 mf/ml; median: 18 mf/ml) overall. Mean *L. loa* microfilaremia was significantly higher in the forest ecosystem than in the savannah (median values: 3469 vs 1357; p = 0.048)) and similar to that in the lakeland (3469 vs 3140; p = 0.18). There was no difference between lakeland and savannah (3140 vs 1357; p = 0.8) (Table 10).

**Table 10:** Intensity of *Loa loa* and *Mansonella perstans* (arithmetic mean microfilaremia) stratified by ecosystem.

*Ecosystem*	Intensity of *Loa loa* microfilaremia		Intensity of *M. perstans* microfilaremia	
	Arithmetic mean mf/ml	Min- Max	Arithmetic mean mf/ml	Min- Max
Savannah	2660	1–17600	34.2	1–400
Lakeland	4626	1–75600	64	1–300
Forest	5742	0–500000	207.7	1–12000
*Grassland*	5859	1–119500	108.7	1–2000
*Mountains*	7777	0–92200	314.8	1–10900
*Inside*	6020	1–500000	313.6	1–12000
*Northern Eastern*	4006	1–83600	19.9	1–200

Likewise, mean *M. perstans* microfilaremia was significantly higher in the forest ecosystem than in the savannah (44 vs 4; p = 0.010) and lakeland (44 vs 0; p = 0.014) (Table 10).

**Figure 4:**
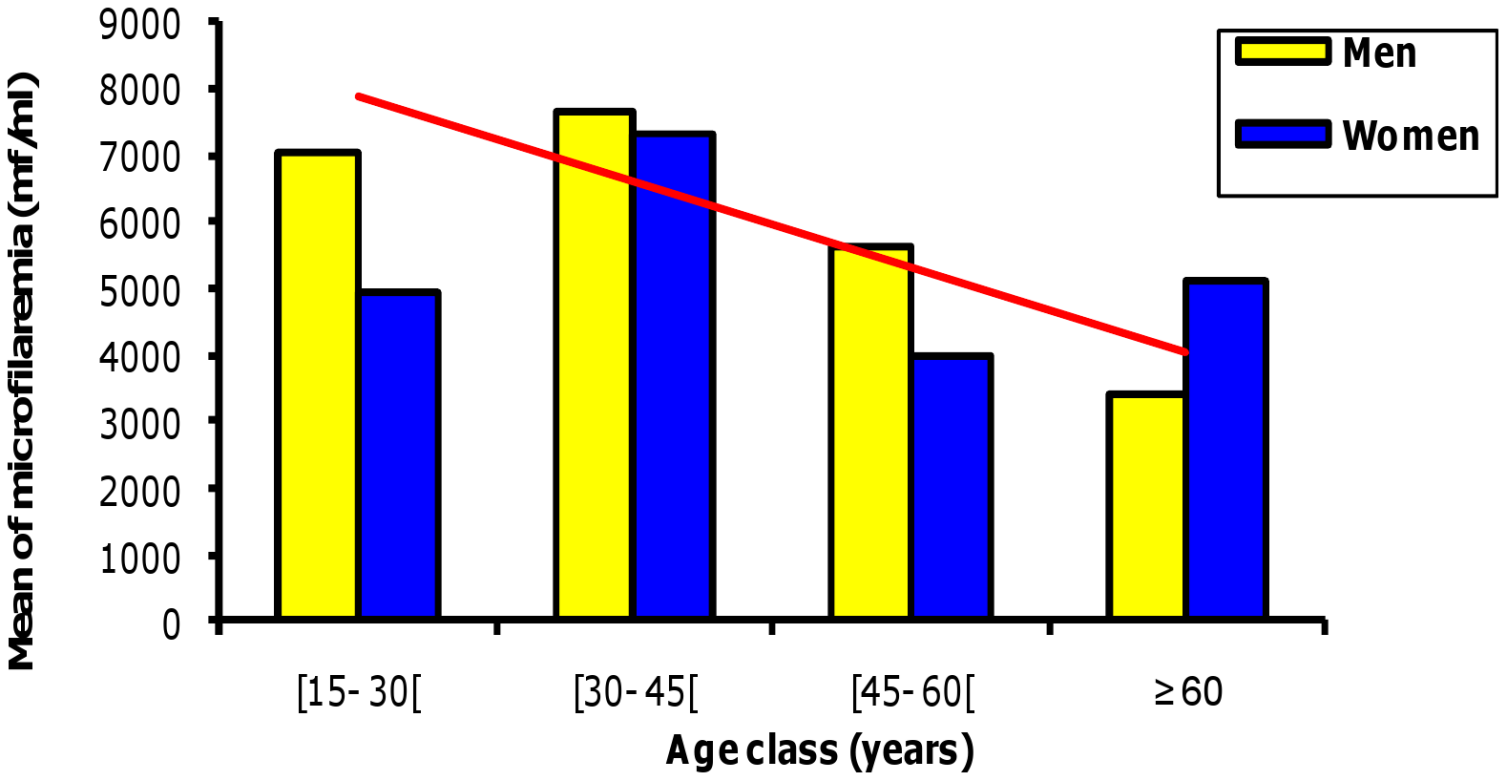
Intensity of *Loa loa* microfilaremia in Gabon according to age and gender.

The intensity of *Loa loa* microfilaremia did not vary with age countrywide (r = 0.249, p = 0.634), while it correlated with age in males (r = 0.915 p = 0.011) but not in females (r = 0.684 p = 0.134) (Figure 4). At the district level, the intensity of *Loa loa* microfilaremia did not vary significantly with age and sex.

### Relationship between the prevalence and intensity of *Loa loa* microfilaremia

**Figure 5:**
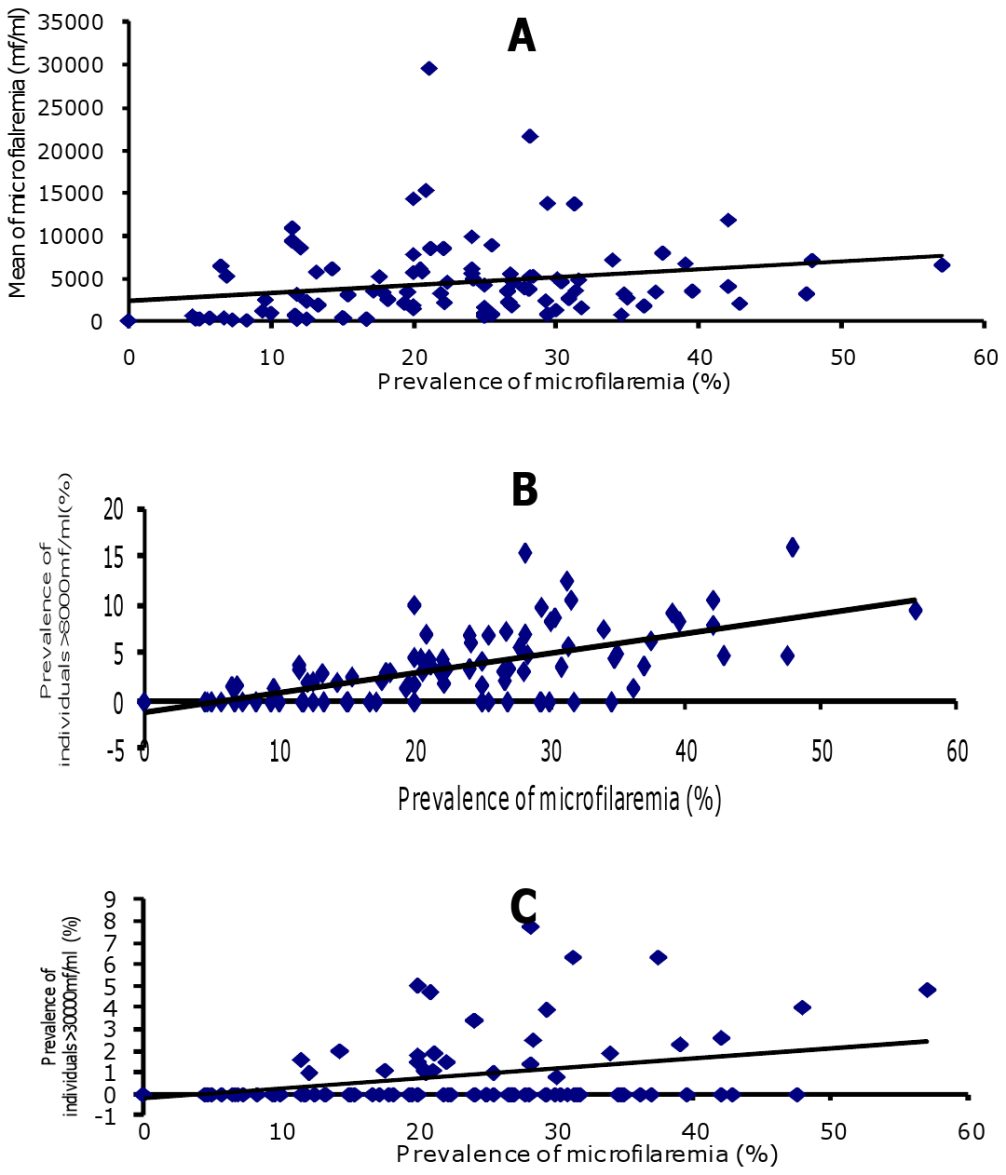
Correlation between the prevalence and intensity of *Loa loa* microfilaremia. **A**. Total studied population. **B**. In individuals with >8000 *Loa* microfilariae/ml. **C**. In individuals with >30 000 *Loa* microfilariae/ml.

The intensity of *Loa loa* microfilaremia (Figure 5A) correlated with the prevalence of microfilaremia nationwide (r = 0.215 p = 0.036) but not at the regional level (r = 0.163, p = 0.675). The intensity of microfilaremia also correlated with the prevalence of microfilaremia >8000 mf/ml (Figure 5B) and >30 000mf/ml (Figure 5C) (respectively r = 0.624, p = 0.0001 and r = 0.319, p = 0.002).

Furthermore, in the subpopulation of individuals with microfilaremia >8000 mf/ml, this relationship was observed in lakeland (r = 0.839, p = 0.001), savannah (r = 0.625, p = 0.027) and forest (r = 0.575, p = 0.0001), while in individuals with microfilaremia >30 000 mf/ml this relationship was only observed in the forest (r = 0.328, p = 0.005).

### Relationship between clinical symptoms and parasitological indices

**Figure 6:**
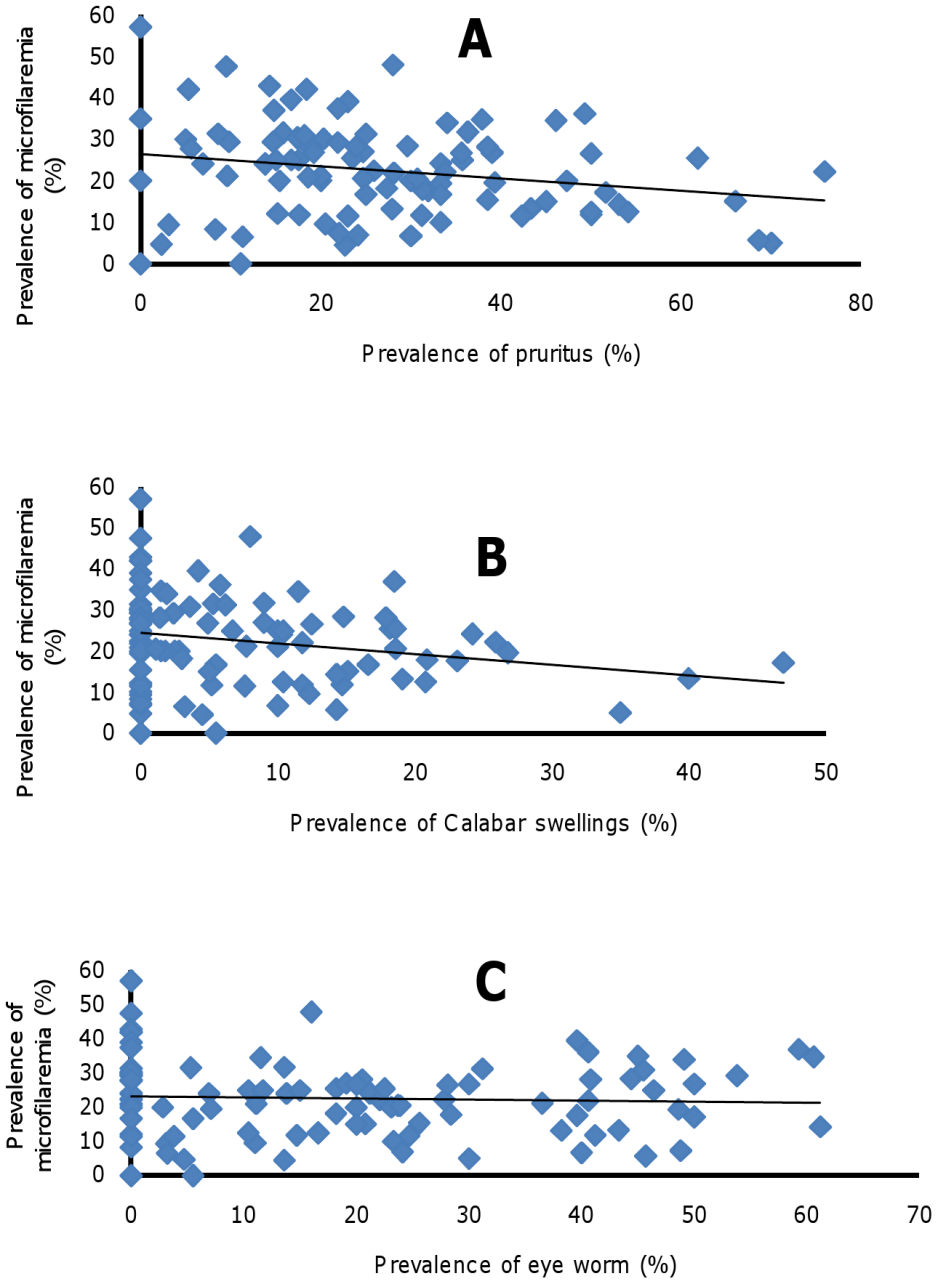
Correlation between the prevalence of *Loa loa* microfilaremia and clinical symptoms. **A**. Pruritus **B**. Calabar swellings. **C**. Eye worm.

The prevalence of pruritis correlated negatively with the prevalence of *Loa loa* microfilaremia (r = −0.219; p = 0.032) (Figure 6A) but not with the intensity of *Loa loa* microfilaria (r = −0.144; p = 0.162) or with very intense microfilaremia (>30 000: r = −0.117; p = 0.255). Similarly, microfilaria >8000 mf/ml correlated negatively with the prevalence of prurits (r = −0.22; p = 0.027). Pruritis was associated with Calabar swellings (r = 0.578; p<0.001) and eye worm (r = 0.425; p<0.001). The prevalence of Calabar swellings (Figure 6B) correlated negatively with the prevalence of *L. loa* microfilaria (r = −0. 220; p = 0.031) but did not correlate with the intensity of microfilaria (r = −0. 061; p = 0.558), microfilaria >8000 (r = −0.185; p = 0.071) or microfilaria >30 000 (r = 0.093; p = 0.370); in contrast, it correlated positively with the prevalence of pruritis (r = 0.578; p<0.001) and eye worm (r = 0.335; p<0.001). The prevalence of eye worm (Figure 6C) did not correlate with the prevalence of microfilaremia (r = −0.05; p = 0.624) or with microfilaremia intensity (r = −0.137, p = 0.182), microfilaria >8000 (r = −0.139; p = 0.178) or microfilaria >30 000 (r = −0.106; p = 0.302), while it correlated positively with pruritis (r = 0.425; p<0.001) and Calabar swellings (r = 0.335; p<0.001). Interestingly, there was no relationship between these three symptoms and the prevalence of *M. perstans* microfilaria (r = −0.146; p = 0.155 for pruritis; r = −0.090. p = 0.385 for Calabar swellings; and r = −0.164; p = 0.110 for eye worm) or the intensity of *M. perstans* microfilaria (pruritis: r = 0.004; p = 0.971; Calabar swelling: r = −0.169; p = 0.100; eye worm: r = 0.182; p = 0.075).

## Discussion

We conducted a large-scale survey of two blood-borne filarial parasites, using direct examination and a concentration technique, in rural populations of 212 villages in Gabon, in order to map their distribution throughout the country, to characterize the modalities of population exposure and to explore the relationship between prevalence and intensity, and between clinical symptoms and parasitological indices.

The overall prevalence rates were 22.4% for *Loa loa* microfilariae, 10.2% for *M. perstans*, and 3.2% for mixed infection. These rates varied across the different ecosystems, the Ogooue River, and the equator. A correlation was found between the prevalence and the intensity of microfilariae, and between clinical symptoms (eye worm, Calabar swelling) and the prevalence of *Loa loa* microfilaremia.

As direct microscopic detection of microfilaria in wet blood films is not very sensitive, we combined two techniques for this survey, namely direct examination of 10 µl of blood (wet film) and prior concentration of 1 ml of blood. If we had used direct examination only, 19.7% *Loa loa* mf carriers and 74% of *M. perstans* carriers would have been missed. Most of these subjects had fewer than 100 mf/ml. Such underestimation may have implications for estimates of the risk of transmission and even for control programmes. Better sensitivity after sample concentration has been reported [14], [30], although this method is more tedious for large-scale surveys. Previous surveys used direct examination with larger volumes (30–50 µl [22], 50 µl [10] or 75 µl [19]).

The prevalence of *Loa loa* microfilaremia was 22.4% overall (up to 57% in some villages) while that of *M. perstans* was 10% (up to 67% in some villages). Gabon is thus a highly endemic country and a zone at high risk of fatal treatment complications. These prevalence rates are similar to those reported in southern Cameroon (up to 38% in the district of Elig-Mfomo) [13] and Equatorial Guinea (27%). This contrasts with Central African Republic (CAR) and Chad, where prevalence is lower (11% and 8.4% respectively). In DRC-Congo, Republic of Congo and Nigeria the prevalence rates range from 1.2% to 97% [13]. It should be noted that these prevalence rates are for specific regions of these countries, whereas our survey covered the whole of Gabon. The prevalence of *Loa loa* remains high in Gabon [15], [28].

*Loa loa* was highly prevalent in the north Equator (>20%), compared to the south (10–20%). Most areas crossed by the Ogooue River from the south-east (its source) to the north-west (towards the Atlantic Ocean) had an *M. perstans* prevalence of more than 10%, while other areas had a prevalence below 10%.

Among the three major ecosystems, forest had a higher prevalence of both parasites than savannah and lakeland. Differences were also seen among the different types of forest, as previously observed in Cameroon [19]. Geographic factors have been implicated in the prevalence of diseases like arteriosclerosis [31]. Sunlight might have a protective effect on some diseases [32], as ultraviolet B radiation stimulates the synthesis of vitamin D, which plays a role in immunity [33]. Geographic factors may influence filarial distribution by affecting the host immune system or the vector. The environment created by Ogooue River may affect the distribution and transmission of *M. perstans*. Although no soil studies around Ogooue River are available, studies in other areas have shown that low-pH soil, low organic soil content, salty soil, and wet soil promote *Culicoides* fly breeding [34], [35] while temperature may affect vector competence [36].

The prevalence of *Loa loa* microfilaremia was influenced by age in both sexes. In some parts of the country the prevalence continued to increase up to 70 years of age, while in others the prevalence appeared to plateau after 60 years. Males tended to be more microfilaremic than females, possibly because men are more exposed to chrysops bites due to their outdoor occupations (hunting, etc.), which become more intense with age, hence the correlation between age and microfilaremia. Genetic factors may also have a role [37]. Furthermore, the negative correlation of the intensity of microfilaremia with age in males may due to concomitant immunity against new incoming infection [38] or natural death of existing microfilariae [39].

In some areas of Cameroon where the general prevalence of microfilaremia exceeds 20%, approximately 5% of individuals have 8000 mf/ml and 2% have more than 30 000 mf/ml [24]. Similarly, in an area with a prevalence of 30%, 9% of carriers had >30 000 mf/ml, while in an area with a prevalence of 40%, approximately 16% of carriers had >8000 mf/ml and 5–6% had >30 000 mf/ml. Therefore, areas with a prevalence of more than 20% are considered to be at a high risk of treatment complications. Such studies have only been conducted in Cameroon [24], [25]. In this study, we observed a positive relationship between the prevalence and intensity of microfilaria. This suggests that the relationship between these two parasitological indices is spatially stable.

Clinical symptoms have also been used to predict the risk of side effects during mass chemotherapy. As previously described, eye worm and Calabar swelling have been found to correlate strongly with prevalence [25]. Photographs of ocular passage of the eye worm were used in previous studies [25]. Whether the lack of photographs in the present study influenced the accuracy of the patients' answers is not known. Yet, in our opinion, the use of photographs would yield a higher prevalence of amicrofilaremic subjects. Another striking observation is the negative correlation of pruritis and Calabar swelling with the prevalence of *Loa loa* but not *M. perstans*. Pruritis is a clinical sign of an allergic reaction. The negative relationship suggests that *Loa loa* filaria may induce desensitization. In Gabon, skin test reactivity against common allergens is low [40], while treatment of helminth infections increases skin test reactivity to mite antigens [41]. Similar observations have been made with *M. perstans* in Ugandan women [42]. A previous study in Gabon showed a high level of polyclonal IgE and *Loa loa*-specific IgG4 in permanent residents [27].

Further investigations are needed to elucidate the relation between filaremia and allergy in Gabon.

In conclusion, we provide a map of *Loa loa* and *M. perstans* microfilaremia in Gabon, and describe important relationships between parasitological indices and clinical manifestations. A clear and spatially uniform relationship was found between the prevalence and intensity of parasitemia. These data should be of use for planning mass chemotherapy.

## Supporting Information

Table S1 **Prevalence and mean of** ***Loa loa*** **and** ***Mansonella perstans*** **microfilaremia in surveyed villages.**
(DOC)

Figure S1 **Questionnaire.**
(PDF)

Checklist S1 **STROBE checklist.**
(DOC)
