# Supplementary material for: Epidemiology of Concomitant Infection Due to Loa loa and Mansonella perstans in Gabon
Source: PLoS Negl Trop Dis. 2011 Oct 11;5(10):e1329. doi: 10.1371/journal.pntd.0001329 (PMC3191124; doi:10.1371/journal.pntd.0001329)
Supplement: Table S1 — Prevalence and mean of Loa loa and Mansonella perstans microfilaremia in surveyed villages. (DOC) [file pntd.0001329.s001.doc]

**Supplementary Table S1**

| **Ecosystems** | ***Villages*** | **Positive *L. loa* / Total examined** | **Prevalence of *Loa loa* microfilaremia** | **Mean *Loa loa* microfilaremia** | **Prevalence *Mansonella perstans* microfilaremia** | **Mean *Mansonella perstans* microfilaremia** | **Population** | **Longitude** | **Latitude** |
| --- | --- | --- | --- | --- | --- | --- | --- | --- | --- |
| **Lakeland** | ***Batanga*** | 2/ 43 | 4.7 | 202 | 4.7 | 54 | 332 | 9.12395 | -1.45432 |
|  | ***Carriere*** | 16/ 38 | 42.1 | 4043 | 18.4 | 51 | 76 | 10.3158 | -0.66953 |
|  | ***Donguila*** | 10/ 21 | 47.6 | 3140 | 33.3 | 74 | 56 | 9.73357 | 0.199579 |
|  | ***Essende_ii*** | 4/ 34 | 11.8 | 153 | 0 | 0 | 88 | 9.59139 | -0.91389 |
|  | ***Kongo*** | 7/ 29 | 24.1 | 6101 | 3.4 | 1 | 212 | 9.4933 | -1.60423 |
|  | ***Malibe*** | 0/ 12 | 0 | 0 | 0 | 0 | 204 | 9.38256 | 0.54824 |
|  | ***Ntchongorove*** | 3/ 32 | 9.4 | 1133 | 0 | 0 | 205 | 9.32177 | -1.91133 |
|  | ***Negue_ntogolo*** | 12/ 99 | 12.1 | 8550 | 0 | 0 | 124 | 10.0659 | -0.97968 |
|  | ***Ntchatanga*** | 13/ 58 | 22.4 | 4546 | 3.4 | 101 | 244 | 10.1207 | -0.82465 |
|  | ***Panga*** | 1/ 20 | 5 | 200 | 0 | 0 | 65 | 10.5568 | -3.22775 |
|  | ***Sette_cama*** | 9/ 68 | 13.2 | 5740 | 0 | 0 | 371 | 9.75151 | -2.51974 |
| **Savannah** | ***Ayem*** | 10/ 27 | 37 | 3370 | 7.4 | 77 | 336 | 11.4367 | -0.11708 |
|  | ***Bikamba*** | 9/ 77 | 11.7 | 681 | 2.6 | 16 | 265 | 11.1197 | -3.18764 |
|  | ***Boutembi*** | 3/ 30 | 10 | 906 | 10 | 5 | 165 | 10.8172 | -2.66545 |
|  | ***Doussala*** | 4/ 58 | 6.9 | 5250 | 6.9 | 51 | 226 | 11.5895 | -2.66 |
|  | ***Douvouli*** | 14/ 55 | 25.5 | 741 | 0 | 0 | 255 | 11.3516 | -2.65536 |
|  | ***Enkassa*** | 8/ 33 | 24.2 | 4904 | 54.5 | 32 | 51 | 13.5352 | -1.34691 |
|  | ***Fera*** | 4/ 62 | 6.5 | 6435 | 3.2 | 10 | 131 | 11.2985 | -2.32584 |
|  | ***Mopia*** | 7/ 35 | 20 | 1808 | 2.9 | 2 | 252 | 13.6077 | -1.81594 |
|  | ***Ossiele*** | 0/ 18 | 0 | 0 | 0 | 0 | 140 | 14.1871 | -0.86299 |
|  | ***Peni*** | 6/ 19 | 31.6 | 4783 | 10.5 | 3 | 221 | 10.4558 | -2.00079 |
|  | ***Saye*** | 1/ 22 | 4.5 | 600 | 0 | 0 | 185 | 14.1773 | -1.73665 |
|  | ***Souba*** | 2/ 24 | 8.3 | 36 | 0 | 0 | 519 | 14.0154 | -1.59431 |
| **Forest** | ***Afoumadzo*** | 25/ 69 | 36.2 | 1722 | 5.8 | 27 | 168 | 12.39 | 0.46343 |
|  | ***Akamsi_effack*** | 20/ 97 | 20.6 | 5739 | 5.2 | 5 | 182 | 11.3905 | 2.26756 |
|  | ***Akoga*** | 4/ 30 | 13.3 | 1854 | 6.7 | 1 | 72 | 10.4935 | 0.86842 |
|  | ***Anguia*** | 11/ 56 | 19.6 | 3373 | 3.6 | 150 | 67 | 11.3945 | 1.61145 |
|  | ***Avazok*** | 15/ 68 | 22.1 | 8503 | 1.5 | 18 | 166 | 11.8293 | 1.53476 |
|  | ***Awoua*** | 24/ 89 | 27 | 4539 | 16.9 | 49 | 743 | 11.6338 | 1.87706 |
|  | ***Ayeguening*** | 16/ 91 | 17.6 | 5189 | 3.3 | 5 | 123 | 11.9168 | 1.92385 |
|  | ***Agricole_centre*** | 15/ 51 | 29.4 | 13807 | 17.6 | 243 | 168 | 10.2594 | 0.04685 |
|  | ***Akok*** | 9/ 21 | 42.9 | 2027 | 28.6 | 71 | 272 | 9.73819 | 0.519373 |
|  | ***Andem*** | 10/ 34 | 29.4 | 753 | 11.8 | 10 | 480 | 9.92997 | 0.355076 |
|  | ***Asseng_assala*** | 12/ 51 | 57.1 | 6538 | 38.1 | 97 | 100 | 10.1474 | 0.4942 |
|  | ***Ayeme_agoula*** | 11/ 35 | 31.4 | 3563 | 34.3 | 44 | 76 | 9.97245 | 0.43037 |
|  | ***Bandi*** | 8/ 40 | 20 | 14324 | 22.5 | 91 | 109 | 11.2438 | -1.84423 |
|  | ***Banga*** | 23/ 66 | 34.8 | 3150 | 10.6 | 35 | 128 | 12.429 | -1.29011 |
|  | ***Baposso*** | 11/ 52 | 21.2 | 8500 | 0 | 0 | 400 | 12.134 | -2.08392 |
|  | ***Bikondome*** | 12/ 67 | 17.9 | 3323 | 7.5 | 6 | 295 | 11.7504 | 2.23194 |
|  | ***Bissobinam*** | 6/ 20 | 30 | 1269 | 35 | 119 | 60 | 12.4553 | 0.50453 |
|  | ***Bissok*** | 19/ 90 | 21.1 | 29648 | 2.2 | 4 | 151 | 11.57 | 1.22186 |
|  | ***Botosso*** | 9/ 26 | 34.6 | 711 | 11.5 | 21 | 95 | 13.0913 | -1.69838 |
|  | ***Dibotsa*** | 12/ 25 | 48 | 7066 | 24 | 424 | 112 | 10.7901 | -2.17735 |
|  | ***Dibwangui*** | 2/ 30 | 6.7 | 361 | 0 | 0 | 270 | 11.5796 | -2.10333 |
|  | ***Douano/_nyali*** | 12/ 54 | 22.2 | 2110 | 1.9 | 2 | 151 | 11.2781 | -2.60074 |
|  | ***Doubou*** | 7/ 29 | 24.1 | 9885 | 0 | 0 | 91 | 10.9122 | -1.77167 |
|  | ***Doussoueoussou*** | 8/ 53 | 15.1 | 351 | 3.8 | 54 | 255 | 10.8788 | -3.03624 |
|  | ***Ebel_abanga*** | 40/ 133 | 30.1 | 4959 | 20.3 | 103 | 800 | 10.4818 | -0.27385 |
|  | ***Ebomane*** | 4/34 | 11.8 | 3125 | 0 | 0 | 440 | 12.0616 | 2.16762 |
|  | ***Ekata*** | 3/ 41 | 7.3 | 102 | 4.9 | 55 | 630 | 14.295 | 0.67584 |
|  | ***Ekouk_chantier*** | 16/ 38 | 42.1 | 11825 | 5.3 | 100 | 708 | 10.3508 | -0.1503 |
|  | ***Emone*** | 7/ 23 | 30.4 | 4603 | 47.8 | 81 | 375 | 9.59771 | 0.83709 |
|  | ***Engoungoume*** | 7/ 61 | 11.5 | 10900 | 13.1 | 130 | 528 | 11.1952 | 0.222 |
|  | ***Etakanyambe*** | 7/ 49 | 14.3 | 6086 | 8.2 | 12 | 342 | 12.9576 | 0.53645 |
|  | ***Eyouga*** | 7/ 22 | 31.8 | 1544 | 9.1 | 402 | 187 | 13.7764 | -1.55593 |
|  | ***Grand_itoumbi*** | 14/ 72 | 19.4 | 2064 | 2.8 | 79 | 186 | 14.112 | 1.14892 |
|  | ***Hendje*** | 11/ 41 | 26.8 | 5486 | 9.8 | 52 | 258 | 13.3897 | 0.26857 |
|  | ***Hevegab*** | 5/ 18 | 27.8 | 3861 | 0 | 0 | 712 | 10.3933 | -0.01586 |
|  | ***Iwatsi*** | 7/ 32 | 21.9 | 3200 | 15.6 | 130 | 130 | 11.8892 | -1.24244 |
|  | ***Junckeville*** | 7/ 20 | 35 | 2663 | 35 | 113 | 164 | 11.2123 | -0.04555 |
|  | ***Keri*** | 9/ 32 | 28.1 | 3700 | 16.1 | 61 | 468 | 10.3638 | -0.74359 |
|  | ***Kouagna*** | 2/ 35 | 5.7 | 352 | 2.9 | 3 |  | 10.634 | -1.09793 |
|  | ***Koumameyong*** | 17/ 55 | 30.9 | 2618 | 10.9 | 376 | 270 | 11.8667 | 0.21224 |
|  | ***La_scierie*** | 11/ 55 | 20 | 7794 | 1.8 | 3 | 138 | 13.1072 | 0.703 |
|  | ***Lambarene_kili*** | 7/ 29 | 24.1 | 5585 | 6.9 | 250 | 362 | 10.3705 | -1.55154 |
|  | ***Lebagny*** | 13/ 65 | 20 | 5675 | 20 | 158 | 252 | 12.5547 | -1.37649 |
|  | ***Loango*** | 6/ 33 | 18.2 | 2501 | 6.1 | 54 | 305 | 10.8621 | -2.72273 |
|  | ***Lolo_1*** | 23/ 81 | 28.4 | 5253 | 4.9 | 110 | 126 | 11.7153 | 0.27548 |
|  | ***Loubomo*** | 17/ 64 | 26.6 | 3469 | 3.2 | 5 | 233 | 10.6332 | -3.2175 |
|  | ***Makatamangoye*** | 10/ 40 | 25 | 531 | 5 | 3 | 130 | 13.7206 | -0.15917 |
|  | ***Massika*** | 9/ 43 | 20.9 | 15315 | 27.9 | 66 | 424 | 10.3685 | -0.82622 |
|  | ***Mayibouth*** | 6/ 39 | 15.4 | 3033 | 5.1 | 10 | 99 | 13.1086 | 1.13424 |
|  | ***Mazingo*** | 12/ 41 | 29.3 | 2327 | 4.9 | 1 | 300 | 14.0879 | 1.37649 |
|  | ***Mbegho*** | 19/ 48 | 39.6 | 3500 | 8.3 | 33 | 135 | 12.3185 | -1.07441 |
|  | ***Mbine*** | 34/ 87 | 39.1 | 6725 | 16.1 | 83 | 168 | 10.2666 | -0.51128 |
|  | ***Mbomo*** | 7/ 73 | 9.6 | 2462 | 5.5 | 155 | 318 | 11.487 | 1.81245 |
|  | ***Medoumou*** | 26/ 102 | 25.5 | 8868 | 6.9 | 9 | 280 | 11.5116 | 1.92473 |
|  | ***Mekouma*** | 7/ 28 | 25 | 889 | 0 | 0 | 234 | 14.074 | 0.82843 |
|  | ***Mela*** | 4/ 20 | 20 | 1440 | 30 | 21 | 61 | 10.2586 | 0.59489 |
|  | ***Mikongo_2*** | 7/ 26 | 26.9 | 1728 | 7.7 | 800 | 276 | 11.7561 | -0.32359 |
|  | ***Mougouango*** | 2/ 12 | 16.7 | 150 | 0 | 0 | 65 | 13.6112 | -2.0104 |
|  | ***Moukabou*** | 11/ 39 | 28.2 | 21659 | 23.1 | 234 | 473 | 10.1372 | -1.60486 |
|  | ***Moukoumbi*** | 18/ 88 | 20.5 | 6073 | 27.3 | 341 | 270 | 12.8993 | -1.1231 |
|  | ***Moyol*** | 5/ 16 | 31.3 | 13740 | 0 | 0 | 55 | 13.9791 | -0.83204 |
|  | ***Ndambi*** | 18/ 53 | 34 | 7155 | 15.1 | 1836 | 165 | 13.2026 | -0.76779 |
|  | ***Ndjokaye*** | 6/ 48 | 12.5 | 2304 | 10.4 | 20 | 142 | 13.7359 | -1.73863 |
|  | ***Ndjole*** | 20/ 71 | 28.2 | 5185 | 28.2 | 801 | 292 | 12.2905 | -1.28306 |
|  | ***Nsimi_esseng*** | 12/ 48 | 25 | 4209 | 12.5 | 13 | 112 | 11.3643 | 2.13278 |
|  | ***Nzingui*** | 12/ 45 | 26.7 | 2183 | 17.8 | 1407 | 292 | 11.6412 | -2.35194 |
|  | ***Okala*** | 15/ 60 | 25 | 1595 | 3.3 | 23 | 240 | 11.6522 | 1.02446 |
|  | ***Okok*** | 11/ 64 | 17.2 | 3466 | 17.2 | 10 | 255 | 11.6753 | 2.09658 |
|  | ***Olounga_ii*** | 3/ 24 | 12.5 | 201 | 66.7 | 80 | 132 | 13.5375 | -1.17026 |
|  | ***Omoy*** | 3/ 26 | 11.5 | 9400 | 3.8 | 12 | 255 | 11.6753 | 2.09658 |
|  | ***Otala*** | 3/ 18 | 16.7 | 241 | 0 | 0 | 568 | 13.8183 | -1.02152 |
|  | ***Paris_bifoun*** | 12/ 32 | 37.5 | 7963 | 18.8 | 164 | 184 | 10.3719 | -0.3357 |
|  | ***Rinanzala*** | 6/ 40 | 15 | 381 | 0 |  | 285 | 11.6944 | -3.29972 |
