# Supplementary material for: Epidemiology of Concomitant Infection Due to Loa loa and Mansonella perstans in Gabon
Source: PLoS Negl Trop Dis. 2011 Oct 11;5(10):e1329. doi: 10.1371/journal.pntd.0001329 (PMC3191124; doi:10.1371/journal.pntd.0001329)
Supplement: Figure S1 — Questionnaire. (PDF) [file pntd.0001329.s002.pdf]

MINISTERE DE LA SANTE PUBLIQUE
……………………….
SECRETARIAT GENERAL
……………………….
DIRECTION GENERALE DE LA SANTE
……………………….
DIRECTION GENERALE ADJOINTE CHARGEE DES PROGRAMMES ET SERVICES NATIONAUX
……………………….

REPUBLIQUE GABONAISE
Union – Travail – Justice

FORMULAIRE ENQUÊTE FILAIRES

ENQUETEUR : ………………………………………………………………………… **DATE : / / /**

N° IDENTIFICATION : …………………………………………………………………………………………….

NOM et PRENOM : ………………………………………………………………………………………………..

Accord pour le dépistage **SIGNATURE :** ………………………

CARACTERISTIQUES SOCIO-DEMOGRAPHIQUES :

Age : ______________ Sexe : ___________

Nombre de personnes vivant dans le foyer : _______

Lieu de naissance : ______________ Nationalité : ______________

Depuis combien de temps habitez-vous à …………………….. : ______________

Statut matrimonial : Marié(e) Veuf Divorcé(e) Concubinage Célibataire

Nombre de partenaires/ épouses / co-épouses : Aucun 1 2 3 Plus

Nombre d'enfants à charge : ______________ Age du dernier enfant : ______________

Niveau d'éducation : Jamais scolarisé Etudes primaires
Etudes secondaires Etudes universitaires

Profession et/ou occupation principale : ____

ANTECEDENTS MEDICAUX

Scarification : OUI / NON

Tatouage : OUI / NON

Avez-vous déjà été opéré ? : OUI / NON si OUI, quelle année ? _______________

De quoi ? ___________________

Avez-vous déjà reçu des injections durant les 6 derniers mois ? : OUI / NON

Avez-vous déjà reçu une transfusion sanguine ? : OUI / NON, si OUI, date de la dernière : ___________

Avez-vous déjà été hospitalisé ? : OUI / NON si OUI, nombre de fois : ______________________

Motifs : ____________________________

Nombre d'accouchements : _________ A domicile : ______Dans une structure sanitaire : _______

Avortements : OUI / NON si OUI, combien ? _______________

QUESTIONS RELATIVES AUX FILAIRES

Avez-vous ou avez- vous eu au cours des 12 derniers mois, ou avant, les symptômes suivants :

- Passage oculaire de filaires OUI / NON
- Des œdèmes douloureux des membres: OUI / NON
- Des démangeaisons : OUI/ NON
- Des lésions cutanées type nodules: OUI / NON
- Des arthralgies ou arthrites : OUI / NON
- Un hydrocèle : OUI / NON
- Un éléphantiasis : OUI / NON

QUESTIONS D'ORDRE GENERAL

Avez-vous récemment présenté les symptômes suivants :

- Toux nocturne: OUI / NON
- Fièvres fréquentes : OUI / NON
- Pertes (vaginales et/ou urétrales) : OUI / NON
- Douleurs abdominales/ Rate/ Foie : OUI / NON
- Troubles neurologiques : OUI/ NON, si OUI, lesquels : Epilepsie, Polynévrite, Paralysie, Autres
- Ganglions : OUI / NON si OUI, localisation : …………………………………………….

HABITUDES DE VIE

Etes-vous chasseur ?: OUI / NON , si OUI, pratiquez-vous : La chasse de proximité La chasse profonde

Quels animaux

Comment : Par le filet/ Piège/ Fusil

Consommez-vous du gibier ?: OUI / NON, Si OUI, lequels ? Singe Rat Palmiste Ecureuil Gazelle Antilope Porc Epic Chauves-souris

Cuisinez- vous le gibier ? OUI / NON, Le dépecez- vous ? OUI / NON

Avez-vous déjà gardé des animaux sauvages ?: OUI / NON si OUI, lesquels ?: ______________________

ENQUÊTE SANTÉ

Bon pour résultat du test sanguin à venir chercher au Centre Médical de votre localité.

ATTENTION, par souci d'anonymat, seul ce document permet de récupérer ses résultats. Tout perte de ce document signifie la perte définitive des résultats.

N° D'IDENTIFICATION : ………………………………………………………….

Signature pour accord :

Le patient qui désire connaître ses résultats doit impérativement signer à la fois ce bon et la fiche qui y est associée. Il faut assurer aux patients le caractère très confidentiel du rendu des résultats. Seul le bon permet de récupérer ses résultats. En cas de perte, tout rendu est impossible.

En cas de positivité, un nouveau prélèvement sera proposé pour confirmation du résultat comme le demande la procédure légale.
