# Supplementary material for: Epidemiology of Concomitant Infection Due to Loa loa and Mansonella perstans in Gabon
Source: PLoS Negl Trop Dis. 2011 Oct 11;5(10):e1329. doi: 10.1371/journal.pntd.0001329 (PMC3191124; doi:10.1371/journal.pntd.0001329)
Supplement: Checklist S1 — STROBE checklist. (DOC) [file pntd.0001329.s003.doc]

STROBE Statement—Checklist of items that should be included in reports of ***cross-sectional studies***

|  | Item No | Recommendation |
| --- | --- | --- |
| **Title and abstract** | 1 | 1. (*a*) Indicate the study’s design with a commonly used term in the title or the abstract:   *Loa loa* and *Mansonella perstans* in Gabon |
|  |  | 1. (*b*) Provide in the abstract an informative and balanced summary of what was done and what was found:   We mapped the prevalence of microfilaremia in 4392 individuals aged between 15-85 years living in 212 villages located throughout Gabon. The overall prevalence was 22.4% for *Loa loa* microfilaremia, 10.2% for *M. perstans* and 3.3% for mixed infection. Correlations were found between the prevalence and intensity of *Loa loa* microfilariae (r = 0.215 p = 0.036). These data confirm the spatial uniformity of the relationship between parasitological indices. |
| Introduction | | |
| Background/rationale | 2 | Explain the scientific background and rationale for the investigation being reported: *Loa loa* and *Mansonnella perstans* are endemic in the central and western African forest block. The first is pathogenic and a major obstacle to the control of co-endemic filariae, as antiparasitic treatment can cause fatal side effects such as encephalitis. Most of these latter studies were performed in Cameroon [24, 25], and their conclusions cannot be extrapolated to other countries because of ecosystem diversity and other specificities |
| Objectives | 3 | State specific objectives, including any prespecified hypotheses:  Map of *Loa loa* and *Mansonnella perstans* prevalence rates. To confirm the linear relationship between the prevalence and intensity of loiasis. |
| Methods | | |
| Study design | 4 | Present key elements of study design early in the paper:  Cross-sectional survey based on microscopic examination of a blood sample |
| Setting | 5 | Describe the setting, locations, and relevant dates, including periods of recruitment, exposure, follow-up, and data collection:  We surveyed rural populations of Gabon between June 2005 and September 2008. The rationale for the study was explained and a one-page questionnaire was administered to all participants. The information collected included demographic data (age, sex, and occupation), geographic location (name of the village, length of residence, department and province) and past medical history (eye worm, Calabar swellings, chronic arthralgia, pruritus, etc.). Individual written consent was required for blood sampling. |
| Participants | 6 | 1. Give the eligibility criteria, and the sources and methods of selection of participants:   Twenty to 30 villages per province were selected at random and individuals over 16 years old having lived in the village for at least one year were sampled with their written informed consent. |
| Variables | 7 | Clearly define all outcomes, exposures, predictors, potential confounders, and effect modifiers. Give diagnostic criteria, if applicable:  Microscopic identification of microfilaria species; comparison of *Loa loa* and *M. perstans* microfilaremia prevalence, intensity of infection, sex, age, ecosystem (forest, savannah, lakeland), and clinical symptoms |
| Data sources/ measurement | 8* | For each variable of interest, give sources of data and details of methods of assessment (measurement). Describe comparability of assessment methods if there is more than one group |
| Bias | 9 | Describe any efforts to address potential sources of bias:  Randomization, logistic regression, systematic use of the concentration technique for microfilaria identification |
| Study size | 10 | Explain how the study size was arrived at:  The required sampling population was calculated on the basis of an estimated filariae prevalence of 5% (using n = ε^2^[p(1-P)]/e^2^; with ε = 1.98 for a confidence interval CI at 95%, e precision = 2% and p = expected prevalence) |
| Quantitative variables | 11 | Explain how quantitative variables were handled in the analyses. If applicable, describe which groupings were chosen and why |
| Statistical methods | 12 | (*a*) Describe all statistical methods, including those used to control for confounding |
|  |  | (*b*) Describe any methods used to examine subgroups and interactions |
|  |  | (*c*) Explain how missing data were addressed |
|  |  | (*d*) If applicable, describe analytical methods taking account of sampling strategy |
|  |  | (*e*) Describe any sensitivity analyses:  *Loa loa* prevalence rates were estimated nationwide and per ecosystem. The Chi2 test and Fisher's exact test were used as appropriate. Minitab 16 software was used to calculate Spearman’s correlation coefficient for the association between parasitological and clinical parameters, and Mann Whitney U test was used to compare mf intensity among group. Univariate crude conditional maximum likelihood estimates of odds ratios (OR) and exact 95% confidence intervals (CI) were determined for each potential risk factor, using STATA software version 9.0 (Stata Corporation, College Station, USA). P values below 0.05 were considered statistically significant. |
| Results | | |
| Participants | 13* | (a) Report numbers of individuals at each stage of study—eg numbers potentially eligible, examined for eligibility, confirmed eligible, included in the study, completing follow-up, and analysed |
|  |  | (b) Give reasons for non-participation at each stage |
|  |  | (c) Consider use of a flow diagram:  4392 individuals. Farmers represented 69.8% of the population, and hunters 10.2%. Around 80% of individuals were surveyed in forest, 10% in savannah an10% in lakeland. |
| Descriptive data | 14* | Give characteristics of study participants (eg demographic, clinical, social) and information on exposures and potential confounders:  In total, 4392 individuals from 15 to 85 years old were enrolled in 212 villages, representing 10.7% of all villages in the country. The sex ratio (M/F) was 0.88. About 58% of individuals were more than 45 years old and 63.9% had spent more than 10 years in their village. Farmers represented 69.8% of the population and hunters 10.2%. Around 80% of individuals were surveyed in the forest area, 10% in the savannah and the lakeland. The reported frequencies of eye worm, Calabar swellings and pruritis were 29.3%, 11.2% and 22.4% (Table 1) |
|  |  | (b) Indicate number of participants with missing data for each variable of interest |
| Outcome data | 15* | Report numbers of outcome events or summary measures |
| Main results | 16 | (*a*) Give unadjusted estimates and, if applicable, confounder-adjusted estimates and their precision (eg, 95% confidence interval). Make clear which confounders were adjusted for and why they were included |
|  |  | (*b*) Report category boundaries when continuous variables were categorized |
|  |  | If relevant, consider translating estimates of relative risk into absolute risk for a meaningful time period:  The overall prevalence was 22.4% for *Loa loa* microfilaremia, 10.2% for *M. perstans* and 3.3% for mixed infection. The prevalence of both filariae was higher in forest than in savannah and lakeland (p< 0.0001). A correlation was found between the prevalence and intensity of *Loa loa* microfilariae (r = 0.215 p = 0.036). There was also a correlation between the overall prevalence of *Loa loa* and the prevalence of microfilaria >8000 mf/ml (r = 0.624; p = 0.000) and >30 000 mf/ml (r = 0.319, p = 0.002). Pruritis and calabar swellings correlated negatively with the prevalence of *Loa loa* microfilaremia (r = - 0.219, p = 0.032; r = - 0.220; p = 0.031 respectivley). |
| Other analyses | 17 | Report other analyses done—eg analyses of subgroups and interactions, and sensitivity analyses  In univariate analysis, males had a significantly higher risk of *Loa loa* infection than females (OR: 2.38, 95%CI: 2.05-2.75, p<00001), and the prevalence of *Loa loa* parasitemia increased linearly with age(p<0.00001) (Table 5A). The prevalence of *Loa loa* microfilaremia was higher in hunters than in farmers and other occupational groups (p<0.04), and higher in individuals with eye worm (p<0.001) and those without Calabar swellings (p<0.014) (Table 5A). Only gender was a risk factor for *M. perstans* microfilaremia, males having a significantly higher prevalence than females (OR: 1.89, 95%CI: 1.54-2.31, p<0.0001) (Table 5B). In multivariate analysis, only age and sex remained significantly associated with *Loa loa* parasitemia throughout the country and within the forest ecosystem (Table 5C and 5D). |
| Discussion | | |
| Key results | 18 | Summarise key results with reference to study objectives:  With a *Loa loa* prevalence of 22.4% overall (up to 57% in some villages) and an *M. perstans* prevalence of 10% (up to 67% in some villages), the whole of Gabon can be considered highly endemic and at a high risk of fatal treatment complications. |
| Limitations | 19 | Discuss limitations of the study, taking into account sources of potential bias or imprecision. Discuss both direction and magnitude of any potential bias:  Eye worm and Calabar oedema have been reported to correlate strongly with parasite prevalence and clinical indices. We found no such correlation. Although photograph was not use, differences between parasitological and clinical indices have been shown in previous studies. |
| Interpretation | 20 | Give a cautious overall interpretation of results considering objectives, limitations, multiplicity of analyses, results from similar studies, and other relevant evidence  Further investigations are needed to elucidate the relation between filaremia and allergy in Gabon. In conclusion, we provide a map of *Loa loa* and *M. perstans* microfilaremia in Gabon, and describe important relationships between parasitological indices and clinical manifestations. A clear and spacially uniform relationship was found between the prevalence and intensity of parasitemia. |
| Generalisability | 21 | Discuss the generalisability (external validity) of the study results |
| Other information | | |
| Funding | 22 | Give the source of funding and the role of the funders for the present study and, if applicable, for the original study on which the present article is based  CIRMF is supported by the Gabonese State, Total-Gabon and Ministère Français de la co-operation. The institution has no role in conducting the study and in preparing the manuscript. |

*Give information separately for exposed and unexposed groups.

**Note:** An Explanation and Elaboration article discusses each checklist item and gives methodological background and published examples of transparent reporting. The STROBE checklist is best used in conjunction with this article (freely available on the Web sites of PLoS Medicine at http://www.plosmedicine.org/, Annals of Internal Medicine at http://www.annals.org/, and Epidemiology at http://www.epidem.com/). Information on the STROBE Initiative is available at www.strobe-statement.org.
